# ‘Strong clinging to objects’: materiality and relationality in Melanie Klein’s *Observations after an Operation* (1937)

**DOI:** 10.12688/wellcomeopenres.16485.1

**Published:** 2021-02-22

**Authors:** Harriet Barratt

**Affiliations:** 1 York St John University, York, UK

**Keywords:** Klein, psychoanalysis, object relations, materiality, patient experience, ‘anxiety-situations’, phantasy

## Abstract

This article presents and analyses a set of notes written by the psychoanalyst Melanie Klein following an operation in 1937. The notes, entitled *Observations after an Operation*, act as a case study of the intersection of psychical, material and social relations as they play out in the immediate aftermath of surgical intervention. Using a close reading method, the article contextualises an analysis of *Observations after an Operation *by linking it to Klein’s wider corpus of theoretical work. It deals in turn with the representation of anxiety mechanisms in the patient experience, drawing upon Klein’s notes on the similarity with ‘anxiety-situations’ in early childhood; with Klein’s changed relation with both external objects and their counterparts in the individual’s mental landscape; with the role of sensation in phantasy, and the connection to bodily pain; with the doctor-patient relationship and the way this is perceived as being embodied in material objects, played out across two dreams experienced by Klein during her recovery; with the emphasis on illness as a form of mourning; and with the creative potential that the experience offers for a renewed structure of object relations. The article concludes that a greater attention to the role and representation of material objects, using psychoanalytic object relations theory as a starting point, can enhance how we collectively understand and assess the psychical impact of healthcare settings upon the patient. It also invites other scholars across the critical medical humanities to consult and analyse the newly available text upon which this article is based.

## Introduction

Materiality, tied up as it is with affective and sensory phenomena, *matters* beyond its tangible limits. Increasingly, the contemporary ‘material turn’ of interdisciplinary humanities scholarship intersects with a renewed emphasis on the intermingling of sociological and psychological experience ( Clarke, 2006, p. 1154; Frosh, 2015, pp. vii–viii; Taylor, 2011, p. 785). This is particularly the case across contemporary theorisations of the ways in which the provision of healthcare takes shape in practice. For example, Victoria Bates (2019) has explored how sensory experience informs the ways in which space and place are perceived by cancer patients; Nik Brown and colleagues (2020) have demonstrated how architectural design reflects, shapes and, sometimes, constrains the treatment of cystic fibrosis patients who are especially prone to air-borne infection; and Nini Fang (2020) has explored the intermingling of psychical and embodied experiences of the anti-immigrant hostile environment, policies which take healthcare as an especial focus. More recently, the COVID-19 pandemic has provoked a spate of public discussion concerned with the sudden, unprecedented re-ordering of domestic and clinical space, the concomitant shift in the sensory realities of care-giving, and the combination of both on an individual’s mental processing. These phenomena work in conjunction (and often in tension) to shape the complex, often contradictory, narratives of an individual grappling to make sense of multiple shifts in social, bodily and emotional experience.

In this article, I present and explore an underanalysed ^1^ piece of autobiographical writing, *Observations after an Operation*, which comprises 15 pages of notes written in 1937 by the psychoanalyst Melanie Klein as she recovered in hospital after an operation. Klein's (1937, p. 6) post-operative notes demonstrate the role of material objects and environments in mental processes at times when one’s physical system is ‘shocked’ through illness or trauma; here, these include a washbasin, cooked fish, flowers, and the view from the window. I aim to demonstrate the extent to which material objects and spaces are bound up with Klein’s conceptions of identification, symbolisation, internalisation and the ‘splitting’ of the object into good and bad ( Klein, 1997b, p. 2), with the healthcare environment which Klein describes giving a particular focus to this exploration. As she later wrote in 1957, ‘under strain from internal or external sources, even well integrated people may be driven to stronger splitting processes’ ( Klein, 1997a, p. 233). Indeed, she proposed that paranoid and depressive anxieties are ‘excessively strong’ in illness ( Klein, 1997d, p. 300), functioning as it does as a revisiting of our ‘earliest internal anxiety-situations’ ( Klein, 1937, p. 8) that can 'shake' our feelings of confidence and security ( Klein, 1998g, p. 391). Klein’s post-operative notes present illness as a restaging of this early experience, much like the analytic setting: the operation and her recovery act as a study in miniature of the process of trauma and reintegration within the wider environment. Key to this process, I will argue, is the experience of illness as a form of mourning as well as one of potential transformation – an extrapolation of Klein’s interest in seeing mourning itself as an illness. Indeed, Claudia Frank (2020, p. 156) sees this piece as a ‘building block’ which allowed Klein to further expand her work on the depressive and paranoid-schizoid positions in the following decade, with the notes’ self-analytic angle forming a crucial part of this role.

It is central to my argument that material objects and spaces give the individual a way to shape and direct these deep, phantasied anxieties ^2^. The sensory, felt, concrete object in Klein’s work and in psychoanalytic theory more widely has too often faded from view, with Christopher Bollas (2009, p. 88) calling for more attention to be given to a material object’s status as a ‘thing-in-itself’ with a ‘specific character’ and ‘its own integrity’. After all, real objects fuel both the process of splitting our own phantasied objects and the subsequent re-integration that comes from ‘reality testing’ relationships to our bodies and external worlds. In *Observations after an Operation*, materiality underpins experience and then recedes as processes of internalisation become manifest in her dreamwork. In effect, for Klein the material object is simultaneously present and absent: a trigger for internalised processes, not a template. Sensory and spatial experience here elicits a complex, multi-faceted set of associations, which both depend upon and supersede the concrete reality of a material environment or object. It is this *constitutive* element – the way that it directs, not dictates, the internal object – that makes materiality so important. In addition, Klein’s emphasis on her post-operative bodily discomfort as a central facet of these processes provides her with a layer of new understanding, namely that the sensory experience of pain is a central part of her concept of ‘memories in feeling’ persisting from very early childhood, explored centrally in her theoretical work ( Klein, 1997a, pp. 180n, 234). *Observations after an Operation* thus not only acts as a fresh piece of source material in relation to Klein’s interest in the interplay between the internal and external object, with the ‘specific character’ ( Bollas, 2009, p. 88) of the material object taking on new importance; it also offers new contextual material on the role of the body within her conception of symbolisation.

This article has a secondary aim. In these notes, it is the expression of Klein’s multiple selves – as a recipient of care, as an infant recreated, and as a psychoanalytic theorist – which together offer a model of object relations specifically located in the spatio-temporality of a patient in recovery within a healthcare environment. As such, it provides a snapshot of how modes of aggression and ambivalence may be played out by patients and staff against the wider backdrop of health and care settings. While the source material in hand would be a rich vein to mine in any case, its particular potency comes from the fact of its writer being a practising psychoanalyst and theorist whose work has fundamentally shaped Western ideas of relationality in the act of care. In this, it echoes writings often positioned as foundation stones of the medical humanities, such as Virginia Woolf’s *On Being Ill*, Audre Lorde’s *The Cancer Journals*, and Anne Boyer’s more recent *The Undying*, in which the thinker is both a patient experiencing somatically complex phenomena and a writer whose craft is directly concerned with representing and interpreting such phenomena. Furthermore, in examining female bodies under intense physical strain – a situation which, as Klein has it, is intimately tied up with the remembered relation to the maternal body – these texts together offer a route towards thinking about how a feminist language for pain, and the mourning that it occasions or recalls, might be said to take collective form ^3^. As such, as well as using this piece ‘to think with’ in relation to these contexts, Klein’s own theoretical work, and the role of self-analysis in the patient experience ^4^, it is my hope that this newly available text may be taken up by other scholars to join (and to complicate) the tacit canon of the critical medical humanities.

## Illness as a restaging of childhood anxiety-situations

### The role of the object

Melanie Klein exhibited an enduring interest in *things* throughout her career – material, concrete objects as well as people (to which the psychoanalytic use of the term ‘object’ more commonly refers) or abstract concepts. This took practical expression in her use of play objects in the clinical analysis of young children, and is a major facet of her conceptualisation of the inner world. In her major work ‘Envy and Gratitude’ (1957), Klein quotes at length from Freud’s comparison of the analyst to the archaeologist, both dealing with ‘material’ as they do:

    [The psychoanalyst’s] work of construction, or, if it is preferred, of reconstruction, resembles to a great extent an archaeologist’s excavation of some dwelling-place that has been destroyed and buried or of some ancient edifice. ( Freud, 2001b, p. 259; cited in Klein, 1997a, pp. 177–78)

The 'stuff' of analysis is, in this sense, a re/constructed ‘artefact’ of thought – but it is also related to the concrete reality of our histories and experiences. This materiality – possessions, spaces and environments – does not exist in a vacuum, but in turn becomes layered with our own projections. As Klein (1998b, pp. 213, 211) wrote in 1929, for ‘every child’ ‘things represent human beings, and therefore are objects of anxiety'; damage to external things can seem to constitute a 'rent in the fabric of the world'.

A concern with the interplay between the ‘real’, external object and the ‘internal object’ which takes shape through phantasy, and how the two intertwine in origin and effect, is thus at the very heart of Klein's theoretical writings: 'there is no instinctual urge, no anxiety situation, no mental process which does not involve objects, external or internal; in other words, object-relations are at the *centre* of emotional life' ( Klein, 1997e, p. 53, emphasis in original). The relationship between the external and internal object in Kleinian theory is not straightforward, however, and must be dealt with carefully. While she acknowledges that external objects and people have ‘at one time contributed to [the] development’ of their counterpart internal objects in any one given individual, she is clear that ‘we must on no account identify the real objects with those which children introject’ ( Klein, 1998d, p. 155). In illustration, she cites the example of a small boy whose internalised, persecutory parental representations sit ‘in the sharpest contradiction to the real love-objects, the parents’, who are in fact ‘unusually kind and loving’. What, though, is taking place in this complex translation from ‘real love-object’ to internalised phantasy, which in turn re-mobilises our relations with others? I turn now to the fifteen pages of *Observations after an Operation* to explore this question in detail.

### Anger and anxiety

Klein underwent gall bladder surgery in London in July 1937, having moved there from Berlin in 1926. We know little more than this about the location or nature of the procedure itself, as no appointment books for that year survive. The operation took place against a backdrop of mourning: her son Hans had died in a climbing accident three years earlier, in April 1934. It was an accident that her daughter Melitta Schmideberg widely proclaimed to be suicide, though this has never been proven ( Grosskurth, 1986, p. 215). Klein's biographer thought that Klein was herself in a 'state of manic depression' during the months that followed, with 1937 being the 'crisis and turning point in Klein's mourning’ ( Grosskurth, 1986, pp. 218, 234).

Klein's first reactions upon waking from the anaesthetic are 'anger, dissatisfaction with the world, persecution' (p. 1); ‘what had been done while I was unconscious was at least in some aspects, because this came out again later on, an attack and injury and had stirred distrust’ (p. 2). The operation initially cuts off Klein’s relationship with external objects, both people and things: ‘I found it very difficult to take interest in anything in these first three or four days’ (p. 2). She is told by the nurses that patients often 'knock everything off' their bedside tables in their immediate, post-operative state of anger and perceived threat (p. 1) – a lashing out at their material surroundings which serves to create the disorder felt both mentally and in the body. Klein conceives of this chaos as a material location, a space 'somewhere' in which she is 'lost' and from which she cannot 'get out' (p. 2).

This necessarily subjective inner object world cannot be tested in the same way as the 'tangible and palpable object-world', meaning that its phantastic nature is self-reinforcing ( Klein, 1998c, p. 346). Only by repeated reality testing in the objective exterior of the ‘real’ world, Klein suggests (following Freud [1934]), can we build a healthy mode of relating between the inner and outer worlds, with material objects and other people helping us to do so. In illness, a time when the body's sensory mechanisms are threatened, the ability to take part in reality testing may be hampered or distorted by drugs, exhaustion, fear, or disorientation. Instead, a fragmentation between subject and environment occurs, and Klein tries ‘to regain relations which had been broken off [by the operation]’ (p. 3).

These attempts are themselves fraught with ambivalence. While Klein’s associations with her external surroundings run more deeply than usual, they are also strangely divorced from her newly insistent inner world. Seeing the sun shining on the brick wall outside her window on the fourth day of recovery, for example, she felt ‘very pleased […] but discovered that I tried to strengthen these feelings of pleasure, suddenly realizing that this whole sunshine on the wall was absolutely artificial and untrue to me’ (p. 3). This confusion and distress is projected onto her material environment in the form of antipathy: she takes a ‘great dislike to the hospital’ (p. 7) and wants to leave early, though this proves impossible from a practical point of view.

Klein’s (1998f, p. 220) 1930 paper ‘The Importance of Symbol Formation in the Development of the Ego’ had by this time already introduced the idea that it is anxiety that ‘sets going the mechanism of identification’, which is in turn, via acts of symbolisation, fundamental to object attachments: ‘it is by way of symbolic equation that things, activities, and interests become the subject of libidinal phantasies’. By 1946, Klein (1997b, p. 3) felt the need to be explicit about her primary focus as being ‘predominantly from the angle of anxieties and their vicissitudes [in relation to objects]’, as opposed to, for example, her colleague Ronald Fairbairn’s emphasis on ego-development. Her trajectory to this point, though, echoes her own theoretical emphasis on the cyclical nature of anxiety and its impact, acting as it does as an obstacle and an impetus in turn. As Lyndsey Stonebridge (1998, p. 39) writes, ‘anxiety has an ambiguous status in Klein’s discourse, both motivating her theoretical conclusions and checking them at significant points’.

### ‘Memories in feeling’

*Observations after an Operation* is a case study in miniature of Klein’s personal and theoretical relations to these iterative, non-linear processes of the mechanisms of anxiety in a physically and sensorially charged hospital setting. Most notably, the experience of being ill or in recovery is presented in Klein’s notes as a return to the primary, pre-verbal anxiety and danger situations experienced in infancy. Klein's surgeon himself volunteers a similar opinion:

    [He] seemed to take great interest in some of the psychological aspects which I discussed with him, and […] said, quite spontaneously and before I gave him such details, that he feels sure that extremely early fears are stirred by an operation, that it takes one back into quite early times, and that, in his view, to recover from an operation is more determined by mastering it psychologically than physically. (pp. 5–6)

The surgeon visualises a movement backwards, with an operation ‘tak[ing] one back’. In a foreshadowing of her thinking in *Envy and Gratitude*, Klein’s (1997a, p. 234) explicit link between the operation and the ‘reviv[al]’ of ‘fundamental situations’ from early childhood instead represents the experience as a *restaging*, a repetition that succeeds despite defence systems:

    I am convinced that what is called a ‘shock to the system’ is a revival of earliest internal anxiety-situations, due to what is felt to be an attack from without and within through the operation, and internal discomfort and pain reviving the early fears of internal persecution. (p. 8)

The phantastic nature of Klein’s reactions to this ‘shock to the system’ is akin to what she calls 'memories in feeling', those deep, almost unreachable layers of experience which come before (and go beyond) language ( Klein, 1997a, pp. 180n, 234). She directly aligns her changing relations to external objects in the days following her operation both with early childhood and with the experiences of patients in analysis:

    I had the feeling, which I have again noticed so often in patients and which stands for a memory, in which a feeling appears as if it had been so in early childhood. (p. 5g)

This emphasis on ‘feeling’ plays on the double valence of sensory and mental experience (we ‘feel’ things both physically and emotionally). It is this interplay between ‘without and within’ (p. 8) which here allows Klein to revive her earliest danger situations. She experiences ‘a great feeling of dependence and anxiety of the nurse’ (p. 7), an observation that recalls Freud’s (2001a, p. 166) belief that anxiety stems from a ‘recognised, remembered, expected situation of helplessness’. Anxiety is thus positioned not only as fear of external loss, but as a threat to one’s deepest unconscious self. As R. D. Hinshelwood (2018, p. 70) puts it in his gloss of Klein’s theories on anxiety, this process is sparked by the precise ‘conditions’ of external experience and internal neurosis in conjunction:

    [W]hen conditions threaten identity, reversion to an expression of the more primitive level of anxiety and mechanisms becomes more apparent. Klein thought that as stress at a neurotic level increases past a certain level, the existence of the ego/self is threatened.

Crucially, however, Klein’s post-operative notes position *re*gression as a form of *pro*gression – a restaging through dreamwork that, rooted in anxiety-induced phantasy, allows her to ‘bring out to consciousness these deep anxieties of persecution inside and outside’, and thus to ‘[regain] my balance, trust in external people, relation with them’ (p. 8). I will return to the transformative potential for relation presented by these processes of anxiety and symbolisation later in this article.

### Sensation, phantasy and pain

Key to this ‘restaging’ process is the way that physical experience sets phantasy in motion and furnishes its content. Klein’s description of the blurring of physical discomfort and psychical pain in *Observations after an Operation* – that ‘strong feeling, connected with the internal discomfort, that all these things [dream phenomena] went wrong inside me’ (p. 4) – supports the idea that one feeds the other in an ongoing exchange of external and phantasied experience. It is Klein’s physical pain that focuses and clarifies the nature of her mental processes, acting as a facilitator and translator:

    Th[e] vivid feeling of internalized processes was of course strengthened and stimulated by the actual discomfort inside me, but on the other hand that just helped me to understand the meaning of this internal discomfort. (p. 5e)

By making bodily (and thus mental) pain temporarily unignorable, the operation foregrounds the cycle of phantasy, mental processing and psychosomatic phenomena, allowing Klein to ‘work with [her]self’ (p. 6). Embodied experience thus fuels and reflects the internal world – each being altered by the other in an ongoing, co-constitutive cycle.

Susan Isaacs (1952, pp. 91–2), a disciple and proponent of Klein’s work, writes in ‘The Nature and Function of Phantasy' – a piece which works in conversation with, and extends, Klein’s work on phantasy – that ‘[t]he first phantasied wish-fulfilment, the first “hallucination” is bound up with *sensation’*; ‘at first, the whole weight of wish and phantasy is borne by sensation and affect’. This is a crucial shift in the conception of the root of object relations. Isaacs places bodily experience at the core of inner life, and, as Klein did, proposes that phantasy is a process that takes place from the earliest times of a child’s life. The interplay between outer and inner worlds is, again, at the heart of this conception of early experience, with external reality ‘coming inside’:

    The earliest phantasies, then, spring from bodily impulses and are interwoven with bodily sensations and affects. They express primarily an internal and subjective reality, yet from the beginning they are bound up with an actual, however limited and narrow, experience of objective reality.

    The first bodily experiences begin to build up the first memories, and external realities are progressively woven into the texture of phantasy. ( Isaacs, 1952, p. 93)

Isaacs lays out here a process of psychical incorporation that echoes, and modifies, ongoing external experience. It is important to note that, in being inextricably tied up with physical sensations and affects, Isaac’s interpretation of phantasy effectively has *material* causes, content and expressions beyond its inherent symbolism. We can read *Observations after an Operation* as an early source which works in conversation with this ensuing development within object relations theory, in which the body as both an internal and external object becomes the focus and the borderland of the intermingling of experience and phantasy.

## Phantasising the mother-doctor

These revivals of infantile anxiety find their expression in Klein’s profoundly symbolic dreams. They are triggered by objects in the sickroom but quickly internalised – an alteration and transformation which swiftly converts material experience into mental representation. In *Observations after an Operation* she describes two dreams in detail. One focuses on a ‘terrifying’ fish, a ‘horrible creature’ (p. 4), which she explicitly links to her dislike of the hospital’s ‘tasteless’ cooked fish (p. 3). The second features ‘a bathroom with the bath tub turned up, gas blowing up and everything going to bits’, potentially linked to her notes on the hospital nurse’s washbasin (pp. 4–5). The heavy symbolism of these dreams, turning on the vulnerability of her body and the people and objects with which she interacts – particularly with care-givers and spaces of care – gives Klein a way to articulate her amorphous and distressing anxiety.

### The attacker and the attacked: the fish dream

In Klein’s first dream, she sees ‘an enormous fish, which I got frightened of’ (p. 3). It is initially ‘a flat fish like a plaice, but not very much like’ (p. 5c); we could read this as a verbal re-rendering of the dream’s status as an altered landscape (a place which is both ‘like’ and ‘not very much like’ the external reality of the hospital). The dream environment is made up of rocks and waterfalls, with ‘something sinister behind the beauty’ (p. 5b), and an ‘important reason to shut a door against the dangers of this water’ (p. 4). ‘[E]verything seemed to go wrong’ in this dream, with ‘a strong feeling, connected with the internal discomfort, that all these things went wrong inside me’ (p. 4). Body, environment and people-objects combine to create an atmosphere of threat and danger, with the surgical experience sparking an unexpectedly intense response: ‘Now this feeling of these objects being internalized [sic] I never had as strongly as in this dream’ (p. 5e).

Klein’s analysis of this dream focuses on her desire to 'suck this fish', which then turns into 'something very terrifying' (p. 4). Her first associations are with maternal figures. The fish is 'a mother whom I had sucked into me and who had turned into a horrible creature', and it reminds her of her daughter-in-law, Judy, ‘a good and motherly figure’ with protruding teeth, known rather unkindly by her husband as ‘fish-face’ (pp. 4, 5c). The fish in the dream becomes a 'real monster' which 'suddenly jumped at my mouth, putting out some sucking part, as it were sucking my lips' (p. 5c). This representation of an external attack on Klein's body becomes, in her associations (and it is not quite clear whether these were present during the dream or arose during her later, waking analysis), about her 'own oral greed'. She sees the fish's attack as a form of projection linked to 'my similar intense and dangerous attacks on [the mother's] breast with my teeth and intensely sucking mouth' (p. 5c) ^5^.

The anxiety over destroying and being destroyed by external objects here is explicitly linked to food – itself an embodiment of ‘the relation to the good and frightening mother’ (p. 5d). Klein is clear that the dream is sparked by the ‘tasteless’ cooked fish provided by the hospital:

    I became more difficult in the second week, when I discovered that the food was actually tasteless and not only because I [ *deleted*: 'was to blame'] [ *inserted*: 'felt it to be so'], and when, it is true, they also took less care to come in than in the first week. But I took a great dislike to the hospital, so much that I wanted to leave it earlier, taking a nurse with me, but did not do this because it seemed too unpractical. (p. 7)

Food, of course, is an odd kind of material object with an inherently liminal status – in being consumed, food is transformed in form, and is no longer separate from the subject. It is telling in this respect that Klein is reminded by the fish dream of a fit she had as a ten-month-old baby, purportedly ‘because my wet-nurse gave me pastry to eat’ (p. 5d). Food is tied up in this anecdote with love and nurture, but equally with aggression and shame – Klein’s mother ‘had warned [her] not to mention [the fit] to anybody’ (p. 5d) because of the potential connection with epilepsy, still a stigmatised condition in this period. Because of this, the episode has ‘always had a sinister meaning’ (p. 5d) for Klein – the same word she uses to describe the setting of the fish dream, with ‘something sinister behind the beauty’ (p. 5b). The blame accorded to the wet nurse – potentially misdirected, given the unclear link between food and fitting for a baby of that age – is presumably because Klein had not yet been weaned, and was seen to be too vulnerable to incorporate inappropriate material. The pastry represents an excess, an over-reaching of her perceived need and capacity.

Klein notes that ‘it is of course interesting that I should have received the breast by this nurse (who was called rather crazy, but quite a good woman) any time when I wanted it’ (p. 5d). Klein does not dwell on this abundance of milk and responsiveness, but her curiosity is evident. As such, it acts a notable aside to her theoretical work on the early infant-breast relation. The wet nurse’s presence itself as an obstacle between mother and infant was new for the family: in Klein’s unpublished, unfinished autobiography, she stresses that her three older siblings were all breastfed by their mother ( Klein, 1959, p. 7). Klein remarks upon her ‘guilt and fears about the rage and anger and sadism which must have gone along with this fit, including my whole relation to the breast’ (p. 5d). In this retelling of another form of illness, her *petit mal* is characterised as an overflow of difficult, destructive feelings which must be hidden from sight: a cutting off of relations between body and mind, and mother and daughter.

In 1952, Klein wrote that a sense of persecutory anxiety ‘always contributes’ to a baby’s dislike of food that is newly introduced to its diet. The food, Klein (1997c, p. 109) proposed, takes on the ‘bad (devouring and poisonous) aspect of the breast’ that had previously been tempered by the ‘good breast’ which provided milk. The food becomes the persecutor, with the infant feeling attacked and unprotected. In the dream the fish comes alive, only to be converted back into potentially edible material by Klein’s desire to suck it – an action linking it directly to the baby’s ingestion of the mother’s milk. It is the moment of incorporation, or at least the *desire* to incorporate the fish into the body by ‘suck[ing]’, which turns it into a thing of terror in Klein’s dream. Although oral experimentation may be a way of testing and establishing reality – phantasied consumption as a form of healthy relation – Klein (1998a, p. 272) proposes the child’s concern with biting and chewing is fundamentally depressive in that it threatens to destroy external good objects. Here, in Klein’s self-analysis of her own restaging of infantile anxiety, it is only when her object relations become more settled that she can assert more agency as a patient, becoming ‘more difficult’ – a state which here represents a form of psychical healing. The seemingly innocent 'correction to her original wording, which positions Klein as being ‘to blame’ for the tasteless hospital food, itself points to a progression of the writing self in these notes from a state of anxious dependence to a more reality-tested stance ^6^. 

Klein’s description of the ‘fish dream’ thus directly mirrors her own theoretical explorations. The idea of oral ‘greed’ is a central element of her work on the weaning process, written the year before the operation, with its emphasis on internalising the object which is about to be lost:

    [I]n phantasy the child sucks the breast into himself, chews it up and swallows it; thus he feels that […] he possesses the mother's breast within himself, in both its good and in its bad aspects. ( Klein, 1998i, p. 291)

In 1935–36, shortly before her operation, Klein had theorised the infant’s aggression as being projected onto external objects. In the process, the baby ‘conceives of them as actually dangerous – persecutors who it fears will devour it, scoop out the inside of its body, cut it to pieces, poison it’ ( Klein, 1998a, p. 262). Twenty years later, Klein (1997a, p. 181) wrote of the implicit violence of this ‘greed’ in a way which might be said to echo the act of the surgeon’s scalpel, suggesting that the baby aims ‘primarily at completely scooping out, sucking dry, and devouring the breast’. The fear of attack is in part a fear of not being protected, and the fish dream can be read as a representation of the surgeon’s perceived failure to keep her internal organs intact and in their proper place: a ‘Mr. J’ is tasked with keeping ‘certain compartments watertight’ by shutting a door and ‘stem[ming] the tide’, but he lets her down ‘as I feel he has done in reality’ (p. 5a) ^7^. Klein notes that this conflation of father figures is related to ‘a whole chain of assocns [sic] – my painful relation with my father’ (p. 5e):

    In the background, Mr. M., the good surgeon, who had represented the good father but whom I did not trust in the dream material. All this, brought into connection with present and past history, was very strongly in connection with the present, but was all internalized. (p. 5e)

The object which is attacked and which attacks in turn, and the concern with bodily and mental incorporation, have parallels in Klein's work on depressive states and anxiety situations in relation to the mother-daughter relationship, too. In 1929 she had written about what she saw as girls' 'most profound anxiety', their ‘earliest danger-situation’: the anxieties stemming from ‘a sadistic desire […] to rob the mother's body of its contents’. This desire ‘gives rise to anxiety lest the mother should in her turn rob the little girl herself of the contents of her body (especially of children) and lest her body should be destroyed or mutilated’ ( Klein, 1998b, p. 217). Aggression breeds anxiety, which itself breeds a fear of bodily loss or mutilation – a risk which, here, the operation has fulfilled (although we do not know the details of the operation, most gall bladder surgery involves the removal of either the gall bladder itself or of gallstones).

### Chaos, guilt and destructiveness: the bathroom dream

The fear of bodily mutilation encapsulated by the fish dream (‘things [going] wrong inside me’: p. 4) recurs in Klein’s dream the following night of an exploding bathroom. The dream sparks ‘first assocns. [sic] [with] my inside and the blowing up of things there, in connection with the assocns. of bad internal objects who had deserted me’ (p. 5). The body acts as a door into psychical processing: in Klein’s post-operative state, the very real surgical intervention in her abdomen (‘my inside’) sparks these earliest of anxieties. The physical discomfort in her abdomen, her gut – a body part which acts at the border of internal/external objecthood, despite being so intimately tied up with the self (we feel things ‘in our gut’, and are warned by our ‘gut instincts’) – increases Klein’s feelings of persecution from 'bad' internal objects, and vice versa. As Frank (2020, pp. 160–61) notes, the ‘surgical removal of an (albeit small) organ on one level acted as a condensation point for all the injuries and losses suffered’. Klein’s gall bladder here acts as a focal point bringing together material and psychical pain, both at the time of the operation and as ‘memories in feeling’: phenomenological experience feeds psychical experience.

There is, once again, an overtone of self-blame to the feeling of ‘things [going] wrong inside me’, which converts a medical intervention into a morally imbued act of self-destruction:

    [I]n the dream about the bathroom being topsy-turvy, the bath tipped upside down, fire breaking out, water rushing, went exactly on the same lines and again had very strong assocns. with very painful actual experiences, phases in my relation to A. [Arthur, Klein’s husband, whom she had left in 1924] of humiliation, disappointment, pain; which now seemed to connect with *feelings of internal destruction, of having been destroyed internally by me..*. (pp. 5e-5f, emphasis added)

In her 1926 paper ‘The Psychological Principles of Early Analysis’, Klein (1998h, pp. 131–32) had linked the phenomenon of young children’s self-injury directly with the manifestation of guilt. We could understand gall bladder surgery, though evidently not self-imposed in reality, as an experience in which the body becomes in phantasy a tool serving to assist in the expression of guilt stemming from feelings of hate and aggression:

    I have found, especially in very young children, that constantly “being in the wars” and falling and hurting themselves is closely connected with the castration complex and the sense of guilt. ( Klein, 1998h, p. 132)

In the same paper, Klein notes that this guilt is tied up with a tendency to pre-empt the feeling of being punished by the parent:

    I found out that the objects against which [Trude, a two-year-old patient] hurt herself (tables, cupboards, stoves, etc.), signified to her (in accordance with the primitive infantile identification) her mother, or at times her father, who was punishing her. ( Klein, 1998h, p. 131)

As in the fish dream, this representation of phantasy ties up material objects with feeling oneself to be both the attacked and the attacker – in both cases, it is one’s body which takes the brunt of phantasies of persecution.

It is not only Klein’s own body which comes into problematic focus in the bathroom dream. Following the concern with the maternal breast in the fish dream, Klein writes in *Observations after an Operation* that ‘the destroyed bathroom […] became clear as the inside of my own mother’ (p. 5f). There are striking examples elsewhere in Klein’s writing of bathroom objects which symbolise a woman’s, or more specifically a mother’s, internal organs ^8^. In ‘The Development of a Child’ (1921), Klein (1998e, p. 35) notes that her son Eric (under the pseudonym of ‘Fritz’) conceived of the womb as a ‘completely furnished house’, which ‘was even possessed of a bath-tub and a soap-dish’. The bath-tub is Eric’s fantasy, but for Klein it is a notable detail. In her later analysis of Dick – a troubled four-year-old boy who had ‘practically no special relations with particular objects' – she notes that he ‘discovered the wash-basin as symbolizing the mother’s body, and he displayed an extraordinary dread of being wetted with water’ ( Klein, 1998f, pp. 224, 226). Elsewhere, Klein (1998d, p. 147) speaks of the importance of providing water ‘above all’ other toys in her play analysis with children. And, although Klein does not make this explicit, we could trace a link between the dream metaphor of the mother’s body as a basin or bathtub and her notes on being washed in hospital by the nurse, an anxiety-inducing mother figure:

    I had a great feeling of dependence and anxiety of the nurse – a ridiculous fact was that for a whole week I allowed the nurse to wash my face with soap and warm water, a thing which I thoroughly dislike. It was after a week I objected, and then had it done the way I wanted it. (p. 7)

In this reading, the dream’s heavy symbolism is once again dependent on material, spatial and bodily metaphors, with the text supporting the possibility that these have been triggered by her experiences in hospital. This link between the material and the maternal (and, here, the medical) is in line with Klein’s (1998b, pp. 213–14) ongoing concern with the process by which ‘[t]he world, transformed into the mother's body, is in hostile array against the child and persecutes him’. In this figuring, the child finds the memory of an earlier maternal relation in the newly-encountered external world: ‘the child’s sadistic phantasies about the interior of his mother’s body lay down for him a fundamental relation to the external world and to reality’ ( Klein, 1989, p. 174).

### Splitting the mother-doctor

The baby’s need to attack the mother as a perceived persecutory object on the one hand, and to be nurtured by her on the other – both tied up with projected loss – is at the root of Klein’s (1997b, p. 2) conception of *splitting* the mother’s breast into ‘a good (gratifying) and bad (frustrating) breast’ ^9^. This splitting into bad and good objects is, paradoxically, part of the process of an integration of external objects’ constituent parts, as Klein later explored in ‘Envy and Gratitude’ (1957). It is in this context that the medical staff plays a significant role in Klein’s representation of her object relations following the operation. She describes two opposing reactions, one regressive and one integrative. Her initial anger upon waking to find herself in pain, to become aware that ‘something [had] been done to her’ (p. 2) while unconscious, stirred distrust. She feels that she has been ‘overwhelmed, cut open, attacked from within’ (p. 6). Her notes contrast these feelings of persecution and ‘deep anxiety situations’ (p. 8) with her ‘actual appreciation that it had all been done so easily, quickly, without my knowing it, my recognition of people’s helpfulness, and my pleasure that it was over, which did not seem to appear at this moment at all’ (p. 1). In particular, she feels ‘very strong gratitude and a very friendly relation to the surgeon’ (p. 5).

The initial perception of the medical staff members as aggressors renders them the source of deep anxiety, despite Klein's cognisance of the fact that they have acted to help her. In effect, Klein sees them simultaneously as 'good objects' offering love and care, and 'bad objects' whose interventions have harmed and disrupted her physical and mental cohesion. Her own regression to much earlier states after the operation restages the process by which the ego 'split[s] itself and its objects [...] in part because the ego largely lacks cohesion at birth, and in part because it constitutes a defence against the primordial anxiety, and is therefore a means of preserving the ego' ( Klein, 1997a, p. 191). In the first few days after the operation, Klein can only perceive those around her as a threat. Her unconscious mind positions the surgeon as a bad object: she refers to ‘Mr. M., the good surgeon, who had represented the good father but whom I did not trust in the dream material’ (p. 5e). These dreams lead her to actively reflect upon her relations with ‘the people who operated on me and whom I tried so much to keep as helpful objects, because otherwise they had just injured and done harm to me’ (p. 5b).

As Klein’s body recovers and her relationships with the world around her are restored, her 'primordial anxiety' and fear of destruction lessen. This allows her to perform the 'gradual integration which stems from the life instinct and expresses itself in the capacity for love' once more ( Klein, 1997a, p. 191). It is important to note that Klein’s post-operative relations to the medical staff are ambivalent, in the Freudian sense of comprising both loving and hateful feelings. If the symbolic breast – the first internal object – is perceived as having been ravaged beyond repair, the split-off parts of the ego cannot be integrated at a later date. In this respect, ‘a certain amount of splitting is essential for integration; for it preserves the good object and later on enables the ego to synthesize the two aspects of it’ ( Klein, 1997a, p. 192). In this reading, by splitting off the medical staff – what she saw as a fundamentally ambivalent act ( Klein, 1998a, p. 287) – Klein is, paradoxically, able to preserve their potentiality as future good objects in preparation for her return to healthy object relating.

## Creative recovery

### Loss and mourning

In Klein’s *Observations after an Operation*, as I have demonstrated, illness and recovery can be a way to think and reflect, with material objects positioned as ‘things to think *with’.* Paradoxically, the use of materiality as a trigger for this act of self-analysis points consistently to one clear theme: loss. In Klein’s notes, this is experienced variously as anxiety (fear of loss), sorrow (a recognition of what has been lost), and anger (a desire to attribute the loss to somebody or something). Klein’s fear of bodily disintegration and mutilation echoes the child’s grief for the lost milk-giving breast, the first object, from which the child must eventually be weaned:

    The object which is being mourned [by the baby before, during and after weaning] is the mother’s breast and all that the breast and the milk have come to stand for in the infant’s mind: namely, love, goodness and security. All these are felt by the baby to be lost, and lost as a result of his own uncontrollable greedy and destructive phantasies and impulses against his mother’s breasts. ( Klein, 1998c, p. 345)

Both this primary loss and the experience of illness described in these notes are experienced as a kind of mourning – a process of decathexis (the withdrawal of emotional investment in a particular idea or object), whether from the mother or from the displaced healthy self. Indeed, Klein’s ‘realization of a feeling of loss of reality’ after seeing the sunshine on the brick wall is accompanied by ‘a relation with grief and the ways of overcoming grief’ (p. 3):

    The process I experienced in mourning [i.e. in her adult life] was to some extent revived, but much less strongly, and it was more easily overcome. I felt, however, a deep longing for people I had mourned, and grief; and feelings of being hurt were accentuated. I felt my system was ‘shocked’. Various characteristics were similar to those in mourning – great sensitiveness, very strong resentment for the slightest thing in which I felt hurt or discomfort – e.g. when I rang the bell and it was not answered – all these things seemed like a psychological assault on me. (pp. 6–7)

Klein sees the adult experience of grief as another direct ‘revival’ of early infantile experience, writing that ‘the child goes through states of mind comparable to the mourning of the adult, or rather, that this early mourning is revived whenever grief is experienced in later life’. To overcome the state of mourning, the child must undertake the ‘work’ of the ‘testing of reality’ ( Klein, 1998c, p. 344). In trying to re-enter the world of objects – the world of relationality – after her operation, Klein is brought up against its opposites: loss and absence, transmuted into a feeling of being ‘assault[ed]’, even by something as minor as an unanswered bell.

The reactions to perceived threat here become blurred, with actual loss, phantasied loss and physical harm intermingling in their affective impact. Such responses are, temporarily, seen as ‘psychotic’ and ‘manic-depressive’ in nature:

    In normal mourning early psychotic anxieties are reactivated. The mourner is in fact ill, but because this state of mind is common and seems so natural to us, we do not call mourning an illness. […] in mourning the subject goes through a modified and transitory manic-depressive state and overcomes it, thus repeating, though in different circumstances and with different manifestations, the processes which the child normally goes through in his early development. ( Klein, 1998c, p. 354)

We ‘do not call mourning an illness’, though it could be understood as such ^10^. Equally, although we do not usually understand illness as a process of mourning, it bears the potential for so many different forms of loss. A patient may experience, or feel that they experience, a loss of freedom, agency and wellbeing; loss of future plans; even the loss of organs or body parts. Perhaps the experience of undergoing a period of illness could even in some circumstances be understood as a ‘modified and transitory manic-depressive state’, from Klein’s description of mourning quoted above. There, it is only by testing one’s object relations – a process wrought with ambivalence – that a process of rebuilding can begin. *Observations after an Operation*, as I will demonstrate, posits that physical recuperation, mental ill health, and mourning may each be seen, in psychical terms, as processes of creative recovery.

### Illness as a ‘potential space’ for transformation

 For the infant, the potential for transformation takes shape in the form of the phantasied mother– hence the persistent concern with her dual role as protector and attacker ^11^. Fear of the loss of this loved object fuels intense anxiety, which translates into aggression. While Klein explores this process in *Observations after an Operation* in minute detail in the analysis of her dreamwork, as explored above, she also describes an alternative, concurrent phenomenon: the way that this time of enforced focus on her own body and mind allows her to ‘work on [her]self’ in a way that ultimately strengthens her relations with the world around her. We could even think of Klein’s portrayal of her period of recovery as a ‘potential space’ – the area between subject and object in which Klein’s colleague, Donald Winnicott (2005a, p. 55), placed the emergence of creativity and exploration.

Klein’s descriptions of her time in hospital are explicitly spatio-temporal. As Klein highlights in *Observations after an Operation* in her representation of post-operative chaos as a threatening space of entrapment (p. 2), the conception of our mental landscape as a space or a boundaried location is implicit in the psychoanalytic concept of an individual's 'inner world'. Klein's vocabulary elsewhere in her theoretical writing ( Klein, 1998c, pp. 362–63) reflects this urge to spatialise and concretise the 'inner world': objects are 'assembl[ed]'and 'built up' 'concretely inside [one]self' to form a world which must be 'rebuil[t]' following an experience of loss. Throwing one back on the internal landscapes and temporalities of the phantasised body as well as the confined space and routine of the bed and sickroom, illness almost takes place ‘out of time’, or at least outside the time of everyday life, with a concomitant shift in one’s relation to oneself.

In this sense, we could understand the world of the sickroom as what Hannah Arendt (1978a, p. 13) calls the ‘small non-time space’ between past and future in which thinking takes place. Arendt’s question on the temporality of thought – ‘When are we when we think?’ – stems from its precursor, *where* are we when we think? ^12^ If illness is a space and time of thinking and examination, though, it is also one in which the temporal nature of recovery and healing come to the fore: a time that is *parallel* to ‘everyday life’, if altered, rather than a ‘non-time’. These ideas are pertinent to a reading of Klein’s *Observations after an Operation* as a symbolic object and space in its own right, acting as it does quite unlike her theoretical work in its autobiographical, note-like form. As a text, it functions as its own ‘break’ from her psychoanalytic corpus, though it is both informed by and informs it in turn. Indeed, the fragmented nature of these notes offer Klein a way to avoid ‘pinning down’ the experience of pain and distress too neatly.

Like the space between analyst and patient, the space of the text and the temporal break of Klein’s operation provide a fertile ground for the re-examination of object relating in a period of ‘strain’ ( Klein, 1997a, p. 133). To complicate this, however, the emphasis on materiality in *Observations after an Operation* tethers it to the immediacy of the care setting, which comprises and informs a delicate web of social and psychical relations. This tension is, I suggest, at the heart of both this piece and of Klein’s wider thinking on internal objects. While ‘we must on no account identify the real objects with those which children introject’ ( Klein, 1998d, p. 155) – and here I see infantile development as standing for the lifelong negotiation of real and introjected objects – it is impossible to disentangle the psychical landscape from the real external setting in which the subject is embedded. Equally, it is impossible to see acts of care provided by others and acts of self-analysis (itself a form of ‘care’) as anything but interlinked – the nurse’s attentive face-washing, for example, becomes entangled with Klein’s own anxiety and sense of being ‘to blame’ for her perceptions of her surroundings:

    I had a great feeling of dependence and anxiety of the nurse – a ridiculous fact [ *inserted*: 'was'] that for a whole week I allowed the nurse to wash my face with soap and warm water, a thing which I thoroughly dislike. It was after a week I objected, and then had it done the way I wanted it. I was a marvellous patient up to this moment, which I was sure was connected with anxiety of the nurse. (p. 7)

The intensity of the anxiety occasioned by the ‘break in life’s continuity’ portrayed here in Klein’s notes – to borrow the words of Winnicott (2005b, p. 131) on trauma – suggests that, in illness, something has been *lost*, or at least put on hold: the chance or ability to relate to external objects and people, subjecthood, agency, physical capacity, independence. I suggest that the ‘break’ of certain time-limited forms of illness and recovery acts as a *temporary* object loss that, while often traumatic, also offers a way to step out of the usual spatio-temporal setting into an area of experience that explicitly calls for one to, in Klein’s words, ‘work with [one]self’ (p. 6). The space of illness thus offers a potential for recovery in psychical as well as physical terms. This is a delicate potentiality, however. Winnicott (2005b, p. 131) stresses that, following trauma, ‘primitive defences now become organized to defend against a repetition of “unthinkable anxiety”’. What may be an inability to recover in the face of catastrophic object loss is here a temporary return to anxiety-situations that may, through what we might think of as *internal* reality testing, ultimately leave object relations strengthened. *Observations after an Operation* demonstrates how, where anxiety is still ‘thinkable’, it may be used as a productive psychical tool. Klein’s notes also help us think about how anxiety and other emotions may equally be *thingable:* experienced as external, material objects.

## Conclusion

*Observations after an Operation* is, I argue, a newly unearthed, important piece of evidence demonstrating the importance of object relations to the patient experience. Its framing of illness and recovery as a potentially transformative restaging of early experience is key: 'everything that can contribute to the elucidation and exact description of the infantile danger-situations is of great value, not only from the theoretical, but also from the therapeutic point of view' ( Klein, 1998b, p. 213). The notes represent the experience of post-operative recovery as not just a physical but a *psychical* process, strongly linked to experiences of internal and external environments. As Klein writes in these notes, her post-operative dreams allow her to explore this cyclical process, to ‘watch step by step the connection between deep internal anxieties, the external experiences that I had been overwhelmed, cut open, attacked from within, and loss of belief in internal and external objects’ (p. 7).

The sensorial content and obdurate materiality of the objects around Klein allows them to act as vital mediators: the sunlight on a wall, a wash-basin, and cooked fish spark heavily symbolic dreams. By working through her associations with these objects in her ‘inner world’ – itself a spatialised rendition of abstract emotions – Klein is able to trace a successful process of thinking, mourning and recovering:

    The work I did with myself showed me how, when I could bring out to consciousness these deep anxieties of persecution inside and outside, I regained my balance, trust in external people, relation with them, while the internal situation had improved. (p. 8)

Klein notes that the revivals of ‘feeling’ (p. 5g) she experienced during her post-operative recovery are directly comparable to the early stages of her life, in which ‘strong clinging to objects’ against the fear of persecution was ‘such a strong feature’ (p. 5g). Although she is here referring specifically to people-objects, these notes also demonstrate how Klein depends upon both the material environment and the body as *matter*, as material to be worked with and worked through in her intensive dreamwork.

*Observations after an Operation* thus points to the centrality of the immediate environment, the body, and pain – phenomenologically, socially and symbolically – in psychical processing. It also underlines the importance of the remembered maternal relationship to a patient’s projections onto their care-givers, from anxiety and guilt to aggression to integration and, potentially, love. An attention to these processes, and to their representation in these notes, simultaneously offers a way to examine the role of the dreaming and writing self in a process of self-analysis, to consider the creative psychical potential inherent to the recovery process, and to demonstrate the importance of the material object to the foundation stones of psychoanalytic theory.

## Data availability

All data underlying the results are available as part of the article and no additional source data are required.

Note on the primary source material: This article builds on archival research carried out in 2017, using the original microfilm of *Observations after an Operation* held at the Wellcome Library (Klein: Unpublished Papers, PP/KLE/C.95; typed manuscript). A scanned copy of the original has since been added to the Melanie Klein Trust website. Klein’s typescript is undated, though the operation she discusses took place in July 1937. Klein added handwritten amendments (which include minor changes in punctuation, insertions and deletions) to the document at a later date, also unknown. For ease of reading, I have incorporated these amendments into the extracts cited in this article without comment, except where they form part of the analysis. I also follow Klein’s original page numbering (pp. 1–8 with additional inserted pages marked 5a-g).

## Notes

^1^ Shortly prior to the submission of this article, a shorter article on *Observations after an Operation* was published in the German-language journal *Luzifer-Amor*; as far as I know, no previous scholarship on this source exists. I would like to acknowledge similarities in our interpretation, which arose independently. As I reference in my article, Claudia Frank helpfully stresses the importance of this source and its self-analytical approach to Klein’s formation of the paranoid-schizoid position and to her ‘implicit view of man as a psychosomatic organization’ ( Claudia Frank, 2020, p. 168). However, while Frank places her study in the context of clinical practice and the development of Kleinian theory, I am primarily concerned with what *Observations after an Operation* can tell us about the potential use of representations of materiality in healthcare as a trigger for psychical processing, and in the use of this approach to the field of the critical medical humanities.

^2^ Klein's conception of 'phantasy' refers to phenomena stemming from the deepest recesses of an infant's unconscious and is thus differentiated from everyday daydreaming or ‘fantasy’. As a development of Freud’s understanding of phantasy, Klein ( *Unconscious Phantasy*, no date) and her followers stress that ‘phantasies *interact reciprocally with experience* to form the developing intellectual and emotional characteristics of the individual’ (emphasis added).

^3^ It is striking that Susan Sontag – whose foundational work on illness Rita Charon referred to ‘a bell that you rang – in the same way that you ring Hippocrates and Osler, you now ring Sontag’ ( Oransky, 2005, p. 468) – tends to use a less personal sweep to theorise illness. Although her personal experience of breast cancer is implicitly suffused throughout her major works *Illness as Metaphor* (1978) and *Aids and its Metaphors* (1988), it fell to her son, David Rieff (2008), to write publicly of the intimate experience of his mother as an ill body within a broader investigation of cultural representations of illness and death.

^4^ As well as the *Handbook of Material Culture*’s ( Tilley *et al.*, 2013, p. 2) description of material objects as things which are ‘good to think [with]’ (itself a repurposing of Claude Lévi-Strauss’ work on totemic animals), this formulation has a parallel in Jo Winning’s (2018) exploration of contemporary case studies as material to ‘think-with’ [sic] in order to address the knotty issues of medical humanities through active engagement with practice.

^5^ This section, with Klein here in the patient-child role, prefigures two dreams described twenty years later by one of her analysands, which she makes central to her work in the essay ‘Envy and Gratitude’ (1957). In these later dreams, a male patient renders Klein as a fish whom he himself ‘suck[s] dry’, which Klein (1997a, pp. 211–12) reads as a way of ‘destroying me and my work out of envy’. The following night, he dreams that he is a small ‘suckerfish’ and that Klein is an ‘old and worn-out pike’ to which he has attached himself. Klein sees this series of identifications as a mobilisation of ‘persecutory as well as depressive anxiety’ which positions her as both an injured and a dangerous object in relation to the analysand.

^6^ This extract also bears directly upon the later work of object relations theorists in the 1950s, who built on the findings around wartime evacuees and separation anxiety to examine the affect of hospitalised children. James & Joyce Robertson (1989, pp. 16–19), for example, who both worked at Anna Freud’s Hampstead War Nurseries and at the Tavistock Clinic, write of the state of quiet despair experienced by a child who has been hospitalised away from their parents. Such a state is sometimes taken for ‘settling in’, but can instead be a ‘danger signal’: ‘Because the child cannot tolerate the intensity of the distress, he begins to make the best of his situation by repressing his longing for the mother who has failed to meet his needs, particularly his need of her as a person to love and be loved by.’

^7^ Neither the Melanie Klein Trust’s Archivist, Dr Jane Milton, nor I could identify this ‘Mr. J’. A potential candidate, fellow psychoanalyst Ernest Jones, was a ‘Dr’, a title which Klein was careful to use where appropriate; Jones is also referred to elsewhere in this papers as ‘E. J.’, so it seems unlikely that this instance refers to him. ‘Mr. J.’, also referred to on page 4 of *Observations after an Operation* as ‘J.O.’ in a description of the same dream, may thus refer to an unidentifiable friend outside the psychoanalytic circle.

^8^ Freud (1920, p. 126), too, sees the presence of water in the dream-world as a symbol of the ‘mother-relation’.

^9^ The splitting of the object into good and bad is not to be confused with the process of fragmentation in Kleinian theory – sometimes also referred to as a process of ‘splitting’ – which occurs in the baby’s regression to an unintegrated state.

^10^ Klein here develops the thinking of Freud (2001c, pp. 243–44), who wrote that ‘although mourning involves grave departures from the normal attitude to life, it never occurs to us to regard it as a pathological condition and to refer it to medical treatment’.

^11^ For more on this concept, see Christopher Bollas’ paper ‘The Transformational Object’ (1987).

^12^ I understand Klein’s representation of the implicitly spatialised ‘inner world’ to be fundamentally different from the thinking of Arendt (1978b, p. 202), who describes the process of converting external reality into thought as one that ‘de-sense[s]’ and ‘de-spatialize[s]’ the original experience.

## References

[ref-1] Arendt H : Between Past and Future: Eight Exercises in Political Thought.New York: Penguin Books.1978a.

[ref-2] Arendt H : The Life of the Mind: Thinking.New York: Harcourt Brace Jovanovich.1978b;1. Reference Source

[ref-3] Bates V : Sensing Space and Making Place: The Hospital and Therapeutic Landscapes in Two Cancer Narratives. *Med Humanit.* 2019;45(1):10–20. 10.1136/medhum-2017-011347 29720481

[ref-4] Bollas C : The Transformational Object.In *The Shadow of the Object: Psychoanalysis of the Unthought Known.*London: Free Association Books,1987;13–29. Reference Source

[ref-5] Bollas C : The Evocative Object World.London: Routledge.2009. Reference Source

[ref-6] Brown N Buse C Lewis A : Air Care: An “Aerography” of Breath, Buildings and Bugs in the Cystic Fibrosis Clinic. *Sociol Health Illn.* 2020;42(5):972–986. 10.1111/1467-9566.13104 32406081

[ref-7] Clarke S : Theory and Practice: Psychoanalytic Sociology as Psycho-Social Studies. *Sociology.* 2006;40(6):1153–1169. 10.1177/0038038506069855

[ref-8] Fang N : Feeling/Being “Out of Place”: Psychic Defence Against the Hostile Environment. *Journal of Psychosocial Studies.* 2020;13(2):151–164. 10.1332/147867320X15869669324256

[ref-9] Frank C : »I was doing a lot of work with myself.« Zu Melanie Kleins unpublizierten Beobachtungen nach einer Operation (1937). *Luzifer-Amor.* 2020;33(65):154–168. Reference Source

[ref-10] Freud S : Symbolism in the Dream.In Stanley Hall, G. (tran.) *A General Introduction to Psychoanalysis.*New York: Boni & Liveright,1920;122–140. Reference Source

[ref-11] Freud S : Formulations Regarding the Two Principles in Mental Functioning (1911).In Riviere, J. (tran.) *Collected Papers.*London: Hogarth Press and the Institute of Psycho-Analysis.1934;4.

[ref-12] Freud S : An Autobiographical Study: Inhibitions, Symptoms and Anxiety; the Question of Lay Analysis and Other Works.Translated by J. Strachey. London: Vintage (The Standard Edition of the Complete Psychological Works of Sigmund Freud, transl. from the German under the general editorship of James Strachey.2001a;20. Reference Source

[ref-13] Freud S : Constructions in Analysis.In *Moses and Monotheism, An Outline of Psycho-Analysis and Other Works.*London: Vintage (The Standard Edition of the Complete Psychological Works of Sigmund Freud, transl. from the German under the general editorship of James Strachey.2001b;23:255–269. Reference Source

[ref-14] Freud S : Mourning and Melancholia.In *On the History of the Psycho-Analytic Movement: Papers on Metapsychology and Other Works (1913-1914).*London: Vintage (The Standard Edition of the Complete Psychological Works of Sigmund Freud, transl. from the German under the general editorship of James Strachey.2001c;14:237–258.

[ref-15] Frosh S : Psychosocial Imaginaries: Perspectives on Temporality, Subjectivities and Activism.Palgrave Macmillan UK (Studies in the Psychosocial).2015. 10.1057/9781137388186

[ref-16] Grosskurth P : Melanie Klein: Her World and Her Work.London: Hodder and Stoughton.1986. Reference Source

[ref-17] Hinshelwood RD : Anxiety and Phantasy.In *The Clinical Paradigms of Melanie Klein and Donald Winnicott.*Abingdon: Routledge,2018;69–75. 10.4324/9780429466922-8

[ref-18] Isaacs S : The Nature and Function of Phantasy.In Riviere, J. (ed.) *Developments in Psychoanalysis.*Hogarth Press,1952;67–121.

[ref-19] Klein M : Observations after an Operation.Wellcome Library (Klein: Unpublished Papers, PP/KLE/C.95). London.1937. Reference Source

[ref-20] Klein M : Melanie Klein’s Autobiography. 1959. Reference Source

[ref-21] Klein M : The Psychoanalysis of Children (1932). London: Virago.1989.

[ref-22] Klein M : Envy and Gratitude (1957).In *Envy and Gratitude and Other Works 1946-1963.*London: Vintage,1997a;176–235. Reference Source

[ref-23] Klein M : Notes on Some Schizoid Mechanisms (1946).In *Envy and Gratitude and Other Works 1946-1963.*London: Vintage,1997b;1–24.

[ref-24] Klein M : On Observing the Behaviour of Young Infants (1952).In *Envy and Gratitude and Other Works 1946-1963.*London: Vintage,1997c;94–121.

[ref-25] Klein M : On the Sense of Loneliness (1963).In *Envy and Gratitude and Other Works 1946-1963.*London: Vintage,1997d;300–313. Reference Source

[ref-26] Klein M : The Origins of Transference (1952). In: *Envy and Gratitude and Other Works 1946-1963*. London: Vintage,1997e;48–56.

[ref-27] Klein M : A Contribution to the Psychogenesis of Manic-Depressive States (1935). In: *Love, Guilt and Reparation and Other Works 1921-1945.*London: Vintage,1998a;262–289.

[ref-28] Klein M : Infantile Anxiety-Situations Reflected in a Work of Art and in the Creative Impulse (1929). In: *Love, Guilt and Reparation and Other Works 1921-1945.*London: Vintage.1998b;210–218.

[ref-29] Klein M : Mourning and its Relation to Manic-Depressive States (1940). In: *Love, Guilt and Reparation and Other Works 1921-1945.*London: Vintage.1998c;344–369.

[ref-30] Klein M : Symposium on Child-Analysis (1927). In: *Love, Guilt and Reparation and Other Works 1921-1945.*London: Vintage.1998d;139–69.

[ref-31] Klein M : The Development of a Child (1921). In: *Love, Guilt and Reparation and Other Works 1921-1945.*London: Vintage.1998e;1–53.

[ref-32] Klein M : The Importance of Symbol-Formation in the Development of the Ego (1930). In: *Love, Guilt and Reparation and Other Works 1921-1945.*London: Vintage.1998f;219–232.

[ref-33] Klein M : The Oedipus Complex in the Light of Early Anxieties (1945). In: *Love, Guilt and Reparation and Other Works 1921-1945.*London: Vintage.1998g;370–419.

[ref-34] Klein M : The Psychological Principles of Early Analysis (1926). In: *Love, Guilt and Reparation and Other Works 1921-1945.*London: Vintage.1998h;128–138. Reference Source

[ref-35] Klein M : Weaning (1936). In: *Love, Guilt and Reparation and Other Works 1921-1945.*London: Vintage.1998i;290–305.

[ref-36] Oransky I : Susan Sontag. *Lancet.* 2005;365(9458):468. 10.1016/S0140-6736(05)17853-2 15724276

[ref-37] Rieff D : Swimming in a Sea of Death: A Son’s Memoir. London: Simon and Schuster.2008. Reference Source

[ref-38] Robertson J Robertson J : Separation and the Very Young. London: Free Association Books.1989. Reference Source

[ref-39] Stonebridge L : The Destructive Element: British Psychoanalysis and Modernism. Palgrave Macmillan UK (Language, Discourse, Society).1998. 10.1007/978-1-349-26721-7

[ref-40] Taylor D : Wellbeing and Welfare: A Psychosocial Analysis of Being Well and Doing Well Enough. *J Soc Policy.* 2011;40(4):777–794. 10.1017/S0047279411000249

[ref-41] Tilley C Keane W Kuechler S : (eds) Handbook of Material Culture. London: SAGE.2013.

[ref-42] Unconscious Phantasy: The Melanie Klein Trust. (Accessed: 9 January 2021). Reference Source

[ref-43] Winnicott DW : Playing: A Theoretical Statement (1971). In: *Playing and Reality.*London: Routledge.2005a;51–70.

[ref-44] Winnicott DW : The Location of Cultural Experience (1967). In: *Playing and Reality.*London: Routledge.2005b;128–39.

[ref-45] Winning J : Learning to Think-with: Feminist Epistemology and the Practice-Based Medical Humanities. *Feminist Encounters: A Journal of Critical Studies in Culture and Politics.* 2018;2(2):20. 10.20897/femenc/3888

