# Supplementary material for: ‘Strong clinging to objects’: materiality and relationality in Melanie Klein’s Observations after an Operation (1937)
Source: Wellcome Open Res. 2021 Feb 22;6:40. [Version 1] doi: 10.12688/wellcomeopenres.16485.1 (PMC8220348; doi:10.12688/wellcomeopenres.16485.1)
Supplement: Peer review history

# Peer review: Reviewer response for version 1

**DOI:** 10.21956/wellcomeopenres.18150.r44176

**Published:** 2021-06-22

**Authors:** Gavin Ivey

**Affiliations:** 1 Discipline of Psychology, College of Health and Biomedicine, Victoria University, Melbourne, Australia

Review of: ‘Strong clinging to objects’: materiality and relationality in Melanie Klein’s *Observations after an Operation *(1937)

 Psychoanalytic historians and detractors, alike, have inevitably mined the interface of personal experience and the professional writing of major psychoanalytic theorists. How does personal experience shape – or skew – theoretical constructions, and how does theory structure and delimit interpretations of personal experience? Melanie Klein devoted her life to understanding the introjective and projective processes whereby the internal world is created from the intercourse of unconscious phantasy and the external world. Perhaps more than most seminal psychoanalytic thinkers, Klein’s writing issued from her personal experience and considerable suffering. In 1937, at the age of 55, Klein underwent gall bladder surgery, a notable event that Klein biographer, Phyllis Grosskurth (1986), ^1^ interestingly fails to mention. Klein, however, carefully noted her postoperative states of mind and recuperation in hospital in an unpublished manuscript, *Observations after an Operation*. ^2^

 Barratt’s paper, only the second published paper devoted to this little known manuscript, is a detailed examination of Klein’s recording of this event. The title, ‘Strong clinging to objects’, derives from Klein’s poignant reference to how her experience of surgery revived early childhood anxieties, “…in which I tried to cling to people, (such a strong feature in my early life, strong clinging to objects), against these fears of inner destruction and persecution” (Klein, 1937, p.12). Klein’s paper is an intriguing piece of close self-analysis, detailing sensations, perceptions, thoughts, memories, and dream imagery prompted by surgical invasion.

 Barratt rightly contextualises Klein’s state of mind prior to the operation, with reference to her protracted mourning of her son, Hans’, death in a climbing accident. I think it is also important to note that Klein was also realistically under attack during this time: her psychoanalyst daughter, Melitta Schmideberg, was pursuing a public vendetta against her, and formidable Freudian critics were vigorously challenging the foundations of her thought. That, together with her tragic loss, would have shaken her internal world and put her objects to the test. 

 Barratt’s contribution, the fruits of a doctoral research project, is essentially a detailed analysis of this self-analysis, as well as a more general consideration of what may happen to our minds as a result of surgical procedures. The long gestation of this paper in the doctoral research process is evident in a carefully crafted piece of textual research. The argument concerns how material objects and spaces are unconsciously invested with meaning and transfigured into textured psychic experiences: "The sensorial content and obdurate materiality of the objects around Klein allows them to act as vital mediators: the sunlight on a wall, a wash-basin, and cooked fish spark heavily symbolic dreams" (p.11). In a containing mind, this transfiguration results in thinkable experience. At the same time anxieties and other powerful emotions may be "thingable", Barratt's neologism for how projected experience assumes the appearance of something tangibly external to us.

 Klein’s writing is distinctively phenomenological; it imagines and portrays the internal world as a visceral, sensation-based reality. Klein feels her way into the infant’s, or the psychotic’s, experiential reality, and evocatively describes what she sees there. She is often accused of privileging phantasy over external reality in developmental experience and the construction of our internal world, a criticism that ignores the fact that external reality only acquires meaning based on its subjective construal in our minds. Though not an explicit focus in Barratt’s paper, the recognition of a dialectic relationship between psychical and material reality is an implicit rejoinder to simplistic critiques of Klein’s work. Barratt is clearly alive to how our bodies *mind*, and our minds *matter*.

 There is much to admire in Barratt’s paper: its assertive but careful argument, detailed attention to Klein’s reported experience, and meticulous background research evident in relevant citations and footnotes. I could find little to critique in this fine piece of psychoanalytic research. I would simply add that there is yet another, but less material, object that Klein clings to in her manuscript. In her paper, Klein writes as though she is a *Kleinian*. This may sound tautological, but the point I wish to make is that Klein is so identified with Kleinian theory that her self-analysis is invisibly structured by this internal object’s possibilities and limitations. It determines her self-understanding while shaping the experience she seeks to understand. That she should cling to it in a time of emotional turbulence, instead of exploring its limits, is no mystery. This, however, is my own stray thought, rather than any significant omission in an excellent paper.

Is the work clearly and accurately presented and does it cite the current literature?

Yes

If applicable, is the statistical analysis and its interpretation appropriate?

Not applicable

Are all the source data underlying the results available to ensure full reproducibility?

Yes

Is the study design appropriate and is the work technically sound?

Yes

Are the conclusions drawn adequately supported by the results?

Yes

Are sufficient details of methods and analysis provided to allow replication by others?

Yes

Reviewer Expertise:

Psychoanalytic psychotherapy; qualitative research methodology

I confirm that I have read this submission and believe that I have an appropriate level of expertise to confirm that it is of an acceptable scientific standard.

## References

[1] : Melanie Klein: Her World and her Work. *Harvard University Press* .1987;

[2] : Observations after an Operation. *Wellcome Library (Klein: Unpublished PAPERS, PP/KLE/C.95)* .1937;

# Peer review: Reviewer response for version 1

**DOI:** 10.21956/wellcomeopenres.18150.r42793

**Published:** 2021-03-25

**Authors:** Rina Kim

**Affiliations:** 1 University of Auckland, Auckland, New Zealand

Harriet Barratt’s article offers a fascinating study on Melanie Klein’s 15 pages of unpublished typed notes, *Observations after an Operation*, ^1^ written when she recovered in hospital after gall bladder surgery in London in July 1937. As Barratt points out, this autobiographical text has not received critical attention except for Claudia Frank’s article published in *Luzifer-Amor *– a German-language journal. We do not often encounter an article that makes us genuinely excited about its findings like this study, and I would like to begin my review by congratulating and thanking the author of this article for drawing our attention to the newly available text written by Klein. In this study, the significance of Klein’s notes are successfully analysed in the context of Klein’s theoretical work, and more importantly, Barratt expands its discussion to the wider setting of healthcare, especially in relation to the significant role of self-analysis in the patient experience. Indeed, as Barratt claims (p. 4), Klein’s text offers a direct account of her own illness and recovery within a healthcare environment and deserves to ‘join the tacit canon of the critical medical humanities’ alongside Virginia Woolf’s *On Being Ill *(1930), Audre Lorde’s *The Cancer Journals* (1980) and Anne Boyer’s *The Undying *(2019).

 According to Esther Sánchez-Pardo (2003, p. 54), ^2^ for Klein ‘mourning is fundamental for keeping our psychological integrity’, and loss is ‘so profoundly inscribed in our psyches that it is always already there’. In other words, the very early psychic and emotional development of the infant and the experience of the loss of the first loved object continuously affect the adult’s psychic processes. What is remarkable about this newly unearthed text by Klein is the ways in which she extends her discussion to physical illness in the healthcare setting, which triggers the restaging of earlier infantile danger-situations in one’s phantasy. As Barratt (p. 11) convincingly shows, the external environments and materials within the context of healthcare play a vital role here, hence making ‘the experience of post-operative recovery as not just a physical but a psychical process’.

 This article is well-researched and offers an excellent close reading of *Observations after an Operation* with useful details from Klein’s notes as well as a helpful link to the unpublished text available online. In general, Barratt (p. 4) successfully situates Klein’s text in relation to her overall theoretical framework, and in the context of an important biographical event, the death of her son Hans in 1934: ‘the operation took place against a backdrop of her mourning’, and the year of 1937 ‘being the “crisis and turning point in Klein’s mourning”’. I understand that Barratt’s main focus lies on what Klein’s text ‘can tell us about the potential use of representations of materiality in healthcare as a trigger for psychical processing’ rather than exploring ‘the development of Kleinian theory’ itself (p. 3). However, it could have been helpful to clearly situate Klein’s text in the context of her famous and related two essays ‘A Contribution to the Psychogenesis of Manic-Depressive States’ (1935) and ‘Mourning and its Relation to Manic-Depressive States’ (1940). R. D. Hinshelwood (1989, p.139), ^3^ for instance, considers the 1935 essay ‘the great theoretical leap’ as Klein moves away from Freud’s binary distinction of mourning and melancholia by addressing the overlooked aspects in his essay such as mania and paranoia in her theory of loss. It would be interesting to read what Klein’s candid self-analysis during her recovery reveals in relation to the development of her crucial theory.

 In this regard, one of the curious omissions in Barratt’s central discussion of Klein’s text is the pervasive sense of fear of persecution and of attack in relation to paranoia as presented in Klein’s notes. According to Klein, in experiencing the loss of the loved one, we experience fluctuations between the depressive state (melancholia) and temporary relief through either returning to the ‘paranoid-schizoid position’[1], or through flight into the manic state.

 As Barratt rightly points out, Klein’s feeling of anger when waking up to find herself in pain is the very first thing Klein addresses. This particular feeling was fuelled by her paranoiac anxiety of ‘something [had] been done to her’ while ‘unconscious’, causing subsequent feeling of ‘dissatisfaction with the world, [and of] persecution’ (Klein, 1937, p. 1). When discussing Klein’s concept of infantile aggression, Barratt (p. 8) explains the mechanism in which the baby’s ‘anxieties stemming from a “sadistic desire”’ to ‘rob the mother’s body of its contents’ which gives rise to a persecution fear that ‘the mother should in turn rob the little girl herself of the contents of her body.’ While Barratt does mention Klein’s gall bladder surgery, it could have been useful to emphasize Klein’s strong feelings of anger and persecution fear, which are triggered by this surgical operation, to her central concept of paranoia.

 Significantly, by opening her notes by expressing her feeling of anger, which she felt ‘was rather striking at that time’ (Klein, 1937, p. 1), Klein acknowledges the innate aggression, which is vital in the mechanism of her theory of paranoia. This concept of paranoia, then, would have strengthened Barratt’s otherwise persuasive reading of bodily pain, material discomfort and external world becoming a trigger for internalised processes. In this vein, it would also have been helpful to introduce and distinguish various forms of reactions to loss Klein developed at the beginning of this article, even if she has not fully developed her theory when she was writing these notes. Similarly, given the centrality of physical illness in its ‘somatopsychic’ nature, as Jean Stora (2007, p.10) ^4^ terms it, and its ‘constitutive element’ (Barratt, p. 3) that directs mental processes, it would be useful to clearly emphasize ‘physical’ illness in the introduction rather than using a more general term ‘illness’.

 As Barratt claims the significance of this unique self-analysis by Klein lies on its framing of physical illness and recovery as a potential space for transformation and creative recovery. This is a ground-breaking reading of the notes, which allows us to see this unpublished text prefiguring her later theory. One’s creative impulse, for Klein, is closely associated with the early child’s aggressive wishes and ‘a destructive attack on or by persecutors — its introjected parents — in phantasy’, and its effort and ‘subsequent attempt to restore the damage to objects’ through the process of reparation (Hinshelwood, 1989, p. 263). While I completely agree with Barrett’s view (p. 11) on ‘the space of the text and the temporal break of Klein’s operation’ providing ‘a fertile ground for the re-examination of object relating in a period of “strain”’, there is scope for more discussion on the aspect of reparation, which is the strongest element of the creative urges and a pivotal part of successful mourning. Indeed, there is a fascinating tension between Klein’s ‘feeling of anger’ expressed and her ‘actual appreciation’ towards the medical staff that ‘[the operation] had all been done so easily and quickly’ (1937, p. 1). Here, while Klein (1937, p. 7) reflects on the event and writes about it, she also *consciously* attempts to repair the damaged objects as experiencing ‘a great feeling of dependence and her anxiety of the nurse’. Klein (1937, p. 8) remarkably concludes, ‘The work I did with myself showed me how, when I could bring out to consciousness these deep anxieties of persecution inside and outside, I regained my balance, trust in external people, relation with them, while the internal situation had improved’.

 The points I made so far, however, are only a few examples that this original study on Klein’s unpublished text opens up. All in all, Barratt’s article offers a new way of engaging with psychoanalytic object relations theory by reading Klein’s notes as a fascinating case study of the psychical impact of healthcare settings upon the patient as well as illness as a potential space for transformation.

 [1] The term ‘position’ designates a configuration of object relations and its characteristic anxieties and defences. Klein uses the term instead of ‘phase’ or ‘stage’ because anyone, as an adult, can find themselves operating within one or other position at any time. Also, although Klein compares paranoid anxieties with melancholia and mania in detail in her essays mentioned here, she did not theorize “paranoid-schizoid position” until 1946. For more discussion about this position, see Kristeva (2001, pp.73-81). ^5^

Is the work clearly and accurately presented and does it cite the current literature?

Yes

If applicable, is the statistical analysis and its interpretation appropriate?

Yes

Are all the source data underlying the results available to ensure full reproducibility?

Yes

Is the study design appropriate and is the work technically sound?

Yes

Are the conclusions drawn adequately supported by the results?

Yes

Are sufficient details of methods and analysis provided to allow replication by others?

Yes

Reviewer Expertise:

• Psychoanalytic theories (Freudian and Kleinian), Memory and Emotion, Cognitive Neuroscience, Contemporary British and Irish theatre, Gender Studies, Medical Humanities, Samuel Beckett

I confirm that I have read this submission and believe that I have an appropriate level of expertise to confirm that it is of an acceptable scientific standard.

## References

[1] : Observations after an Operation. *Wellcome Library (Klein: Unpublished PAPERS, PP/KLE/C.95)* .1937;

[2] : Cultures of the Death Drive: Melanie Klein and Modernist Melancholia. *Duke University Press* .2003;

[3] : A Dictionary of Kleinian Thought. *Free Association Books* .1989;

[4] : When the Body Displaces the Mind: Stress, Trauma and Somatic Disease. *Karnac Books* .2007;

[5] : Melanie Klein. Translated by Ross Guberman. *Columbia University Press* .2001;

# Response

**Published:** 2021-04-05

**Authors:** Harriet Barratt

**Affiliations:** York St John University, UK

Thank you so much for such a generous, considered and useful review! I'm so grateful for all the time you have invested in this. Your suggestions are really helpful, and I will be revising the article with them in mind after receiving a second review, at which point I will respond more fully. Many thanks again.
